# Commentary: 20 years online with “Your Disease Risk”

**DOI:** 10.1007/s10552-020-01356-3

**Published:** 2020-10-17

**Authors:** Graham A. Colditz, Hank Dart

**Affiliations:** Division of Public Health Sciences and Alvin J. Siteman Cancer Center, Department of Surgery, Washington University School of Medicine, Washington University in St. Louis, 660 South Euclid Avenue, Campus, Box 8100, St. Louis, MO 63110 USA

**Keywords:** Application, Commentary, Prevention, Risk assessment, Risk communication, Translational medicine

## Abstract

The Your Disease Risk tool (yourdiseaserisk.wustl.edu) went live to the public in January 2000 and was one of the first personalized health risk assessment sites on the Internet. Its launch marked the culmination of years of work by a large, multi-disciplinary university team whose primary goal was to translate the science on cancer prevention into accurate, engaging, and useful messages for the public. Today, 20 years on, Your Disease Risk has expanded from its initial four cancers to include 18 different tools designed for today’s users. This commentary reviews important moments and lessons learned in the first two decades of Your Disease Risk.

**Electronic supplementary material:**

The online version of this article (10.1007/s10552-020-01356-3) contains supplementary material, which is available to authorized users.

## Introduction

**Fig. 1:**
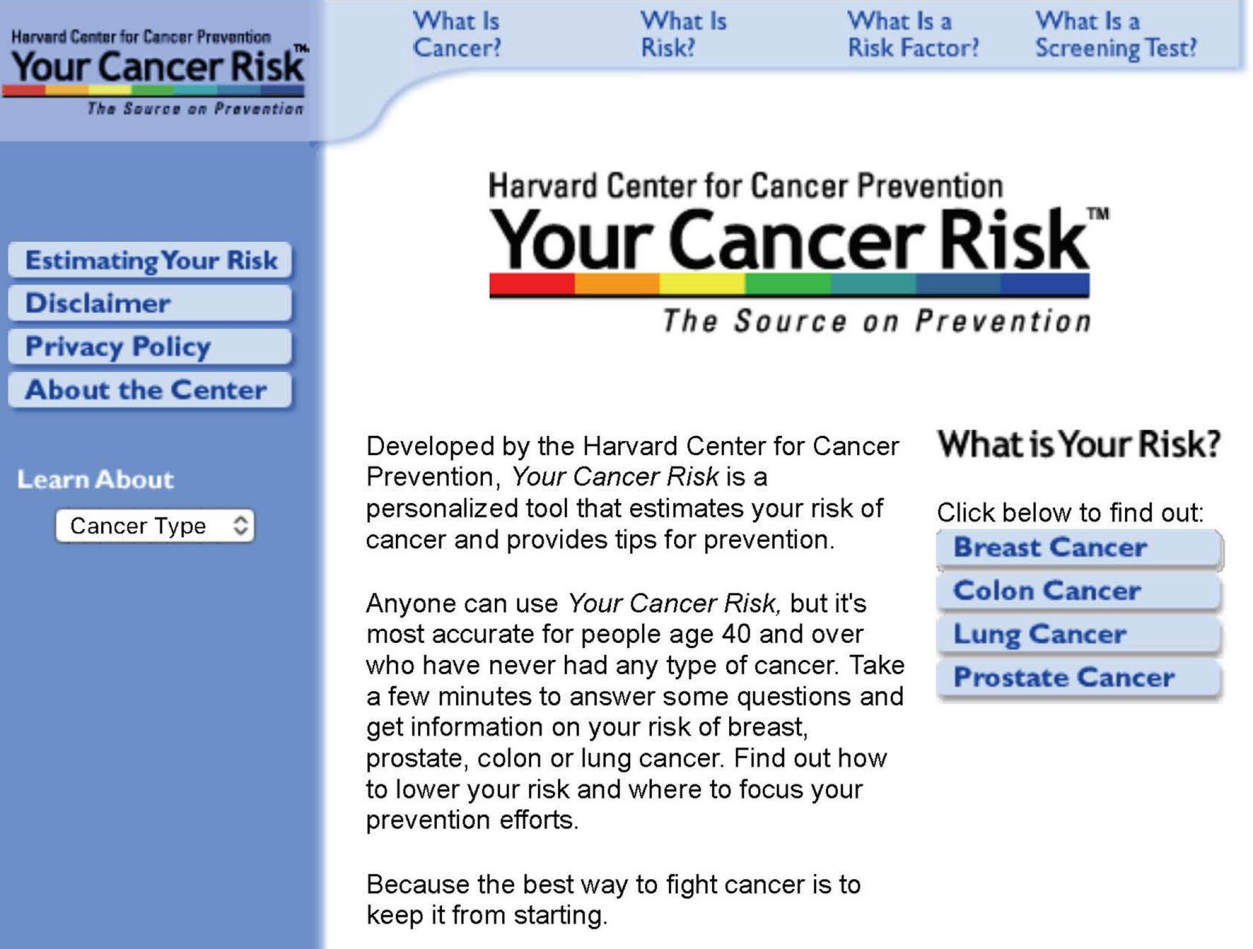
Your Cancer Risk in January 2000

Near the height of the Dot-Com Boom in January 2000—when Y2K was still in the headlines and AOL announced its merger with Time-Warner—we launched one of the first personalized health risk assessment sites on the Internet. What would eventually become Your Disease Risk (yourdiseaserisk.wustl.edu), started out that January as Your Cancer Risk, a tool that provided risk estimates and personal prevention plans for four common cancers: breast, colon, lung, and prostate (Fig. 1). It was the culmination of years of work by a large, multi-disciplinary university team whose primary goal was to translate the science on cancer prevention into accurate, engaging, and useful information and messages for the public [1–3].

**Fig. 2:**
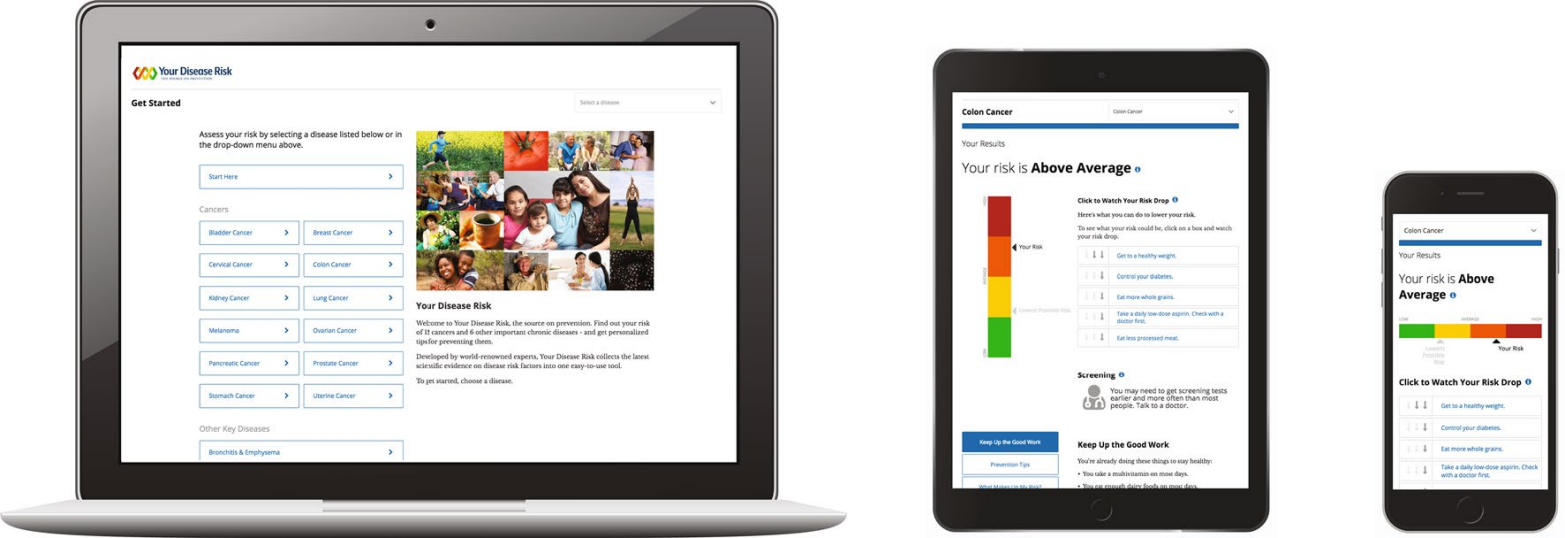
Your Disease Risk in 2020

Today, Your Disease Risk has evolved to meet the needs of modern users as a web app with responsive design (Fig. 2). Its menu has expanded to include 18 different tools offering assessments for diabetes, heart disease, osteoporosis, stroke, chronic obstructive pulmonary disease, and 12 cancers; and it continues to provide a framework for research and development of other risk prediction models [4–6].

This is an overview of select key moments and lessons learned in the first 20 years of Your Disease Risk.

## 2000 —from pencil and paper to the web

Your Disease Risk was first conceived and drafted as a set of pencil and paper quizzes called the Harvard Cancer Risk Index, which had the intent of providing the public with an accurate and easy-to-use way to assess personal risk of cancer (Supplemental Fig. 1) [1]. Its calculations were developed over many months through a process that resulted in consensus agreement on cancer risk factors and their level of evidence and magnitude of association, which is described in detail in Colditz et al. 2000 [1]. In brief, the cancers included in the Harvard Cancer Risk Index accounted for approximately 80% of cancer incidence in the United States, and the group consensus process identified the genetic, environmental, nutritional, lifestyle, and personal health factors that were determined to be established or probable causes of each cancer. To take into account variations in interpretation of evidence, ranges were used to assign the magnitude of association between risk factor and cancer rather than a precise point estimate (e.g., “weak RR 1.2 to < 1.5” and “strong RR 3.0 to < 7.0”). The categories were then translated to risk points for each exposure. These points could then be summed to provide an individual an overall cancer risk score. Using Surveillance Epidemiology and End Results (SEER) data, this score was then presented as a categorical risk compared to the population average risk (e.g., “Much below average risk”) as well as a 10-year absolute risk (e.g., “4 in 1,000”). Validity of the Risk Index was assessed in two large prospective cohorts (Nurses’ Health Study and Health Professional Follow-up Study), with good agreement found for colon cancer between multivariate logistic functions and the grouping approach of the Risk Index [1].

Pilot testing with members of the public, however, revealed an important problem with the basic approach of the Risk Index calculations: a high percentage of those completing the tool summed their risk points incorrectly. It would not matter how good the risk models were or how much time had been spent developing them if we could not deliver accurate numbers to the individuals completing the quizzes. Further iterations did not substantially improve the issue. So, rather than continue to refine the hand calculation options, we decided to move in a radical direction for the time, and one that would determine the life of the tool over the next two decades. We would do calculations for people in real time by translating our pen and paper tool to a dynamic website. It was 1999. Google search was still in beta testing.

Qualitative evaluations of the Risk Index and an early online colon cancer tool (unpublished) (Supplemental Fig. 2) informed a number of initial ideas for the full online version of the new tool [7]. These included providing personalized lists of protective modifiable factors, providing personalized lists of non-modifiable risk factors, and taking into account numeracy and literacy in both risk and message communication.

Based on this, our approach in developing the risk estimate display, a primary function of the tool, was to adopt as simple a method as possible. After detailed consideration, we determined that using a verbal categorical relative risk result combined with a simple graphic would likely provide a level of precision that would meet the needs of users of the online tool. While early versions of the Risk Index and a pilot colon cancer tool had used absolute risk results, absolute risks can often be misunderstood, even in highly educated populations [8], and in A/B tests of the pilot tool, users had preferred the verbal relative risk display to that of numeric absolute risk. Compounding this was the complexity of the absolute risks that would need to be displayed in the tool, especially considering the full range of cancers we would be including. Using today’s SEER data, for example, the average risk of invasive stomach cancer in a 50 year old (all races) is 0.10% (1 in 1,000) over 10 years and 0.28% (2.8 in 1,000) over 20 years [10]. In younger users, and those at lower risk, the frequencies would be smaller and likely even harder for many in the public to interpret correctly. We were concerned that that level of complexity could detract from the overarching goal of the online tool, to motivate healthy behavior change [9, 11]. Therefore, we employed the combined results display of categorical relative risk while also providing options for users to access background information that could help further place disease risk in context.

The first complete version of the online tool contained most of the elements still seen in today’s version, including estimates of disease risk, an accounting of the modifiable and non-modifiable factors that drive a user’s risk result, and the ability to see dynamically how adopting healthy behaviors could lower that risk in the future. This latter “risk manipulation” option remains a cornerstone of the tool’s functionality and has been linked to possible improvement in risk perception accuracy [12] as well as improved response efficacy [13]. The potential disease risk reduction users view when choosing a healthy lifestyle change is based on the magnitude of association assigned during the consensus process to each factor. The risk increase associated with a risk factor is negated, or the risk decrease associated with a protective factor is applied. While this general approach could be refined as the science on behavior change and its impact on risk modification further develops, it has allowed us to provide a broad demonstration to users of prevention’s potential average benefit across multiple diseases and for scores of lifestyle factors.

The use of plain language throughout the tool has been a key part of development from its inception. Assessments have shown good performance on both SMOG reading grade-level (9th grade) and the Suitability Assessment of Materials (SAM) scale (Superior), particularly in comparison to other similar websites and tools [14, 15]. In line with the focus on plain language, we also rebranded the tool from the academic-sounding Harvard Cancer Risk Index to the simpler and more engaging Your Cancer Risk. Although the individual “Your” in the title did not necessarily align with the probabilities of risk estimation, we felt that it did align well with the intent of users of the tool, and other tools like it: to receive an estimate of their disease risk.

After approximately a year of planning, coding, and pilot testing, we launched Your Cancer Risk on 19 January 2000, offering risk estimates and personalized prevention recommendations for breast cancer, colon cancer, lung cancer, and prostate cancer. It proved an immediate success, with 2.7 million hits in its first week alone, more than 80 times the traffic expected (Supplemental Fig. 3). At that time, the site was arguably unique in what it offered, both in its approachable risk estimates but also in its cancer prevention messages, which few other sources had available at that time either online or off.

Six months later, we added eight additional cancers, bringing the total to 12 (Supplemental Fig. 4).

## 2004—expanding with Your Disease Risk—and the 8IGHT WAYS

Most serious and preventable chronic diseases—like cancer, heart disease, and diabetes—share many risk factors. Furthering this message was our inspiration for updating Your Cancer Risk in 2004 to include heart disease, stroke, diabetes, and osteoporosis. The expanded tool was then rebranded to its current name: Your Disease Risk.

This same year, a validation study of selected cancers using two large prospective cohorts was published [16], with results showing well-calibrated risk estimates for the general population. Age-adjusted concordance statistics for individual cancers were colon cancer in men 0.71 (95% CI 0.68–0.74), colon cancer in women 0.67 (95% CI 0.64–0.70), pancreatic cancer in men 0.72 (95% CI 0.67–0.77), and ovarian cancer 0.59 (95% CI 0.56–0.62). A further validation of Your Disease Risk was published in 2015 assessing discriminatory accuracy of the heart disease tool in middle-aged women in a large prospective cohort. The age-adjusted concordance statistic for the model was 0.71 (95% CI 0.69–0.72), a result on par with established cardiovascular disease risk assessments that use more clinical factors in their tools [17].

Your Disease Risk was not primarily developed as a research tool. Its focus, rather, has been the translation of complex information into a free, publicly available website that offers users an organic online experience. While this approach has a number of positive aspects, it has somewhat limited the scope of Your Disease Risk-related research, especially in relation to broader topics of public health. To date, no research has assessed the impact of the tool on, for example, population risk reduction. However, evidence from a small study suggests the tool’s approach may help improve social-cognitive precursors to healthy behavior change, including intentions, response efficacy, and self-efficacy [13].

The 2004 expansion of Your Disease Risk also saw the origination of the 8IGHT WAYS cancer prevention series (8ways.wustl.edu) [18, 19]. Initially added to the site as a static information source for visitors to the tool who may not want to complete a personalized risk assessment, it has itself become an important tool for prevention outreach. Today, there are seven stand-alone 8IGHT WAYS guides available in multiple languages and special editions. In addition to being popular destinations on our cancer center website, the 8IGHT WAYS have also been integrated into worksite wellness programs as well as community outreach campaigns of the Program for the Elimination of Cancer Disparities (Supplemental Fig. 5).

## 2005—adaptations and the Spanish-language Cuidar de su Salud

One overarching goal that has driven many decisions on the direction of Your Disease Risk has been the desire to reach as wide an audience as possible with its important health messages. This has led to the tool’s adaptation across the globe, including in Israel, Canada, Sweden, the Netherlands, Ireland, and the United Kingdom, as well as tailored use within the Unites States, including free-standing kiosks in community-selected settings in Washington, DC [20–22]. In April 2005, we launched our own unique version of Your Disease Risk: The Spanish-language Cuidar de su Salud (Supplemental Fig. 6). Design and content of the tool were tailored based on multiple focus groups with Spanish speakers. The color palette was modified. Selected questions and prevention messages were revised to be more culturally relevant and appropriate, and the overall design space was adjusted to accommodate the sometimes lengthier Spanish text, which maintained the same accessible reading level of the English version. The Spanish tool ran for multiple years and was ultimately archived due to maintenance issues. However, we have sustained the capability to re-integrate Cuidar de su Salud and other translations into Your Disease Risk’s latest design (see below).

## 2009—web traffic and media coverage

Your Disease Risk has had a history of stressing server capacity with its spikes in web traffic. This was especially common in its earlier years when such spikes were hard to predict and cloud hosting as we know it today was still in its infancy. Coverage in major news outlets was directly responsible for most of this traffic [23–27]. These stories primarily described functional aspects of the site, such as the manner in which it worked and its user experience. A 2009 New York Times piece, A Better Health Quiz, however, focused on an aspect of Your Disease Risk that has been a cornerstone of development from day one, and one that still makes it stand out in the modern tech landscape: Its lack of data sharing, financial conflicts of interest, and advertisements (Supplemental Fig. 7) [28].

## 2012—the spin-off Zuum app

Integrating Your Disease Risk into physicians’ offices and hospital waiting rooms had been something we had wanted to do with Your Disease Risk almost from its outset. Even though some early research had used the site in the clinical setting, its design was not optimized for either the time or space constraints of medical offices [12, 29]. Inspired to address this limitation, we developed the iPad app Zuum (zuum.wustl.edu) using the Your Disease Risk framework (Supplemental Fig. 8). Instead of 17 different assessments in a tool designed for a desktop computer, we created a mobile app with a single brief survey that could provide accurate risk estimates—and prevention recommendations—for five key diseases. A research version of the app was pilot-tested in Federally-Qualified Health Centers in the St. Louis area, and its algorithms were adapted for clinic-based studies in Boston [30–32]. The Zuum tool remains available as a stand-alone app and has also been adapted within the most recent update of Your Disease Risk.

## 2018—a major update for modern users

While the science that drives the Your Disease Risk calculations is evaluated and updated on a schedule, the overall design and software of the site had not had a major change since 2004. With so much of the world accessing online content through mobile devices, we saw a need to update Your Disease Risk to a responsive design, so that it would provide modern users an equally satisfying experience whether they were on smartphones, tablets, or laptops. Given the space constraints of mobile screens, the major challenge in doing this was distilling the original content of Your Disease Risk down to core messages while at the same time maintaining accuracy and engagement. After many months of development and testing, the updated site officially launched in January 2018.

## 2019—building on historic traffic

We estimate that Your Disease Risk has had 5 million or more visitors since its launch. While not Facebook or Google numbers, for a public health tool that maintains only part-time staffing, it is nevertheless noteworthy. Admittedly, going from being a unique tool in the early 2000s to today being one of many risk assessment tools, we have been pushed to maintain and build upon our historic visitor numbers. This has prompted us to explore, with the help of our cancer center marketing group, the potential benefits of targeted, paid media campaigns. Such a campaign would have two related goals: to boost prevention message outreach to populations within our center’s catchment area, and to build core web traffic to the tool. A test ad-buy in 2018 showed promise on both fronts, and in 2019, we began a modestly priced 12-month campaign (Supplemental Fig. 9). Even with tight distribution targets within Missouri and Illinois, there was more than a tripling of user traffic to the site in the two weeks after the ads launched, suggesting there could be significant potential for future campaigns and related prevention outreach efforts.

## 2020—key lessons from 20 years of prevention

Spend 20 years with any single project and the highlights (and lowlights) from that period will be too numerous to cover in one short reflection—and that is certainly our experience with Your Disease Risk. Together, though, they have revealed some core tenets that will continue to inform the future of Your Disease Risk and, we hope, others’ related projects. Briefly, here are three.

### Simple and accurate can coexist

For all its complex algorithms developed over months of scientific debate, much of the true success of Your Disease Risk seems to come from its simplicity. Detailed calculations are translated to simple questionnaires that nearly anyone can complete in just a few minutes, and without the need to have lab results or medical records in hand. Likewise, the takeaway messages are easy to understand, practical, and brief. Although this approach can feel foreign to some researchers and others in the medical field, that simplicity can sacrifice little, if any, of the accuracy of a tool and the risk results it delivers [33, 34].

### There is great value in a varied team

It is hard to conceive that Your Disease Risk would have been as successful as it has been—and for as long—if it had not been developed with a multi-disciplinary team. Among those involved, there were epidemiologists, biostatisticians, clinicians, health communication researchers, programmers, designers, writers, and editors. Each brought specific expertise to a tool that had multiple facets but one overarching goal: to communicate accurately and effectively about risk and prevention to the public.

### Well-crafted messages can resonate

Throughout the development and evolution of Your Disease Risk, one of our primary goals has always been to develop prevention-related materials that resonate with the public, and its long-term popularity strongly suggests that that has been a valuable focus. Even in today’s frenetic media and information environment, people seek out quality information about their health. We in public health can offer them compelling and trustworthy messages that can play a role in helping prevent disease and promote health. Because of that, developing and distributing such well-crafted messages should be a priority.

We are honored to have been able to work on this project these past two decades, and with such an accomplished, dedicated, and inspiring group of contributors across multiple institutions. Support from the Siteman Cancer Center, Washington University, Barnes-Jewish Hospital, and the Foundation for Barnes-Jewish Hospital has been essential to the tool’s continued scientific upkeep, evolution, and success.

With a nod to the past 20 years, we look forward with excitement to Your Disease Risk’s future.

## Electronic supplementary material

Below is the link to the electronic supplementary material.

Supplementary file1 (PDF 2424 kb)

## Data Availability

Your Disease Risk code is not publicly available; however, the underlying relative risks used in its calculations can be accessed at: api.yourdiseaserisk.org/about.

## References

[1] Colditz GA, Atwood KA, Emmons K, Monson RR, Willett WC, Trichopoulos D, Hunter DJ (2000) Harvard report on cancer prevention volume 4: Harvard Cancer Risk Index. Risk Index Working Group, Harvard Center for Cancer Prevention. Cancer causes & control : CCC 11 (6):477–488 10.1023/a:1008984432272 10880030

[2] Harvard Center for Cancer Prevention (1996). Harvard report on cancer prevention. Volume 1: causes of human cancer. Cancer Causes Control CCC.

[3] Harvard Center for Cancer Prevention (1997). Harvard Report on Cancer Prevention Volume 2: Prevention of human cancer. Cancer Causes Control CCC.

[4] Marshall AA, Zaccardelli A, Yu Z, Prado MG, Liu X, Miller Kroouze R, Kalia SS, Green RC, Triedman NA, Lu B, Deane KD, Iversen MD, Karlson EW, Sparks JA (2019). Effect of communicating personalized rheumatoid arthritis risk on concern for developing RA: a randomized controlled trial. Patient Educ Couns.

[5] Prado MG, Iversen MD, Yu Z, Miller Kroouze R, Triedman NA, Kalia SS, Lu B, Green RC, Karlson EW, Sparks JA (2018). Effectiveness of a web-based personalized rheumatoid arthritis risk tool with or without a health educator for knowledge of rheumatoid arthritis risk factors. Arthritis Care Res (Hoboken).

[6] Sparks JA, Iversen MD, Miller Kroouze R, Mahmoud TG, Triedman NA, Kalia SS, Atkinson ML, Lu B, Deane KD, Costenbader KH, Green RC, Karlson EW (2014). Personalized risk estimator for rheumatoid arthritis (PRE-RA) family study: rationale and design for a randomized controlled trial evaluating rheumatoid arthritis risk education to first-degree relatives. Contemp Clin Trials.

[7] Emmons KM, Koch-Weser S, Atwood K, Conboy L, Rudd R, Colditz G (1999). A qualitative evaluation of the Harvard cancer risk index. J Health Commun.

[8] Lipkus IM, Samsa G, Rimer BK (2001). General performance on a numeracy scale among highly educated samples. Med Decis Making.

[9] Zikmund-Fisher BJ (2013). The right tool is what they need, not what we have: a taxonomy of appropriate levels of precision in patient risk communication. Med Care Res Rev.

[10] Howlader N, Noone AM, Krapcho M, Miller D, Brest A, Yu M, Ruhl J, Tatalovich Z, Mariotto A, Lewis DR, Chen HS, Feuer EJ, Cronin KA (2020). SEER cancer statistics review, 1975–2017.

[11] Han PK, Hootsmans N, Neilson M, Roy B, Kungel T, Gutheil C, Diefenbach M, Hansen M (2013). The value of personalised risk information: a qualitative study of the perceptions of patients with prostate cancer. BMJ Open.

[12] Emmons KM, Wong M, Puleo E, Weinstein N, Fletcher R, Colditz G (2004). Tailored computer-based cancer risk communication: correcting colorectal cancer risk perception. J Health Commun.

[13] Fowler SL, Klein WMP, Ball L, McGuire J, Colditz GA, Waters EA (2017). Using an internet-based breast cancer risk assessment tool to improve social-cognitive precursors of physical activity. Med Decis Making.

[14] Cortez S, Milbrandt M, Kaphingst K, James A, Colditz G (2015). The readability of online breast cancer risk assessment tools. Breast Cancer Res Treat.

[15] Kaphingst KA, Zanfini CJ, Emmons KM (2006). Accessibility of web sites containing colorectal cancer information to adults with limited literacy (United States). Cancer Causes Control CCC.

[16] Kim DJ, Rockhill B, Colditz GA (2004). Validation of the Harvard cancer risk index: a prediction tool for individual cancer risk. J Clin Epidemiol.

[17] De Vito KM, Baer HJ, Dart H, Chiuve SE, Rimm EB, Colditz GA (2015). Validation of a risk prediction tool for coronary heart disease in middle-aged women. BMC Womens Health.

[18] Dart H, Wolin KY, Colditz GA (2012). Commentary: eight ways to prevent cancer: a framework for effective prevention messages for the public. Cancer Causes Control CCC.

[19] Wolin KY, Dart H, Colditz GA (2013). Eight ways to stay healthy after cancer: an evidence-based message. Cancer Causes Control CCC.

[20] Lophatananon A, Usher-Smith J, Campbell J, Warcaba J, Silarova B, Waters EA, Colditz GA, Muir KR (2017). Development of a cancer risk prediction tool for use in the uk primary care and community settings. Cancer Prev Res (Phila).

[21] Elias S, Grooters HG, Bausch-Goldbohm RA, van den Brandt PA, Kampman E, van Leeuwen FE, Peeters PH, de Vries E, Wigger S, Kiemeney LA (2012). The Dutch cancer society cancer risk test. Ned Tijdschr Geneeskd.

[22] O'Neill E, Davies-Cole, J., Ellis, E., (2013) Community health kiosks in district of Columbia: a health disparities information system. Paper presented at the 141st APHA Annual Meeting, Boston, MA, November 6, 2013

[23] Revill J (2004) Harvard can now asses your health risks via the internet. The Guardian, July 4, 2004. https://www.quitsmokingandstartexercisingbeforeitstoolate.com

[24] Parker-Pope T (2006) Website tallies your risk of disease and tells you what you can do about it. Wall Street J, October 31, 2006

[25] Voelker R (2000). Quick uptakes: online risk assessment expands. JAMA.

[26] Favorite health resources (2008) New York Times, September 29, 2008

[27] Health: Finding a Diagnosis (2006) US News and World Report, November 12, 2006

[28] Parker-Pope T (2009) A better health quiz. New York Times, March 27, 2009

[29] Weinstein ND, Atwood K, Puleo E, Fletcher R, Colditz G, Emmons K (2004). Colon cancer: risk perceptions and risk communication. J Health Commun.

[30] Colditz G, Izadi S, Dart H, Waters E, James A (2017). Integrating a health risk assessment mobile app into diverse primary care settings—a Pilot Project. Cancer Epidemiol Biomarkers Prev.

[31] Haas JS, Baer HJ, Eibensteiner K, Klinger EV, St Hubert S, Getty G, Brawarsky P, Orav EJ, Onega T, Tosteson AN, Bates DW, Colditz G (2017). A cluster randomized trial of a personalized multi-condition risk assessment in primary care. Am J Prev Med.

[32] Baer HJ, Schneider LI, Colditz GA, Dart H, Andry A, Williams DH, Orav EJ, Haas JS, Getty G, Whittemore E, Bates DW (2013). Use of a web-based risk appraisal tool for assessing family history and lifestyle factors in primary care. J Gen Intern Med.

[33] Liu Y, Colditz GA, Rosner BA, Dart H, Wei E, Waters EA (2018). Comparison of performance between a short categorized lifestyle exposure-based colon cancer risk prediction tool and a model using continuous measures. Cancer Prev Res (Phila).

[34] Rifas-Shiman SL, Willett WC, Lobb R, Kotch J, Dart C, Gillman MW (2001). PrimeScreen, a brief dietary screening tool: reproducibility and comparability with both a longer food frequency questionnaire and biomarkers. Public Health Nutr.

