# Supplementary material for: Commentary: 20 years online with “Your Disease Risk”
Source: Cancer Causes Control. 2020 Oct 17;32(1):5–11. doi: 10.1007/s10552-020-01356-3 (PMC7796866; doi:10.1007/s10552-020-01356-3)
Supplement: Supplementary file 1 — Supplementary file1 (PDF 2424 kb) [file 10552_2020_1356_MOESM1_ESM.pdf]

Electronic Supplemental Material - 1

Commentary: 20 Years Online with "Your Disease Risk"

Colditz and Dart

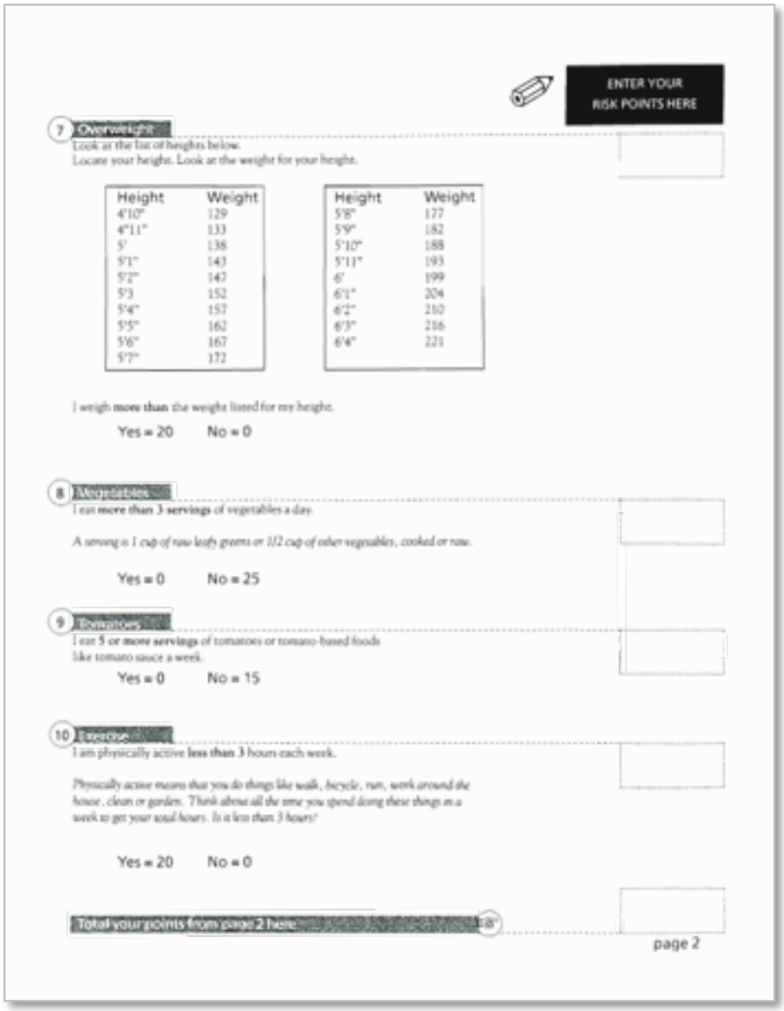

ENTER YOUR RISK POINTS HERE

7 Overweight

Look at the list of heights below.
Locate your height. Look at the weight for your height.

Height	Weight
4'10"	129
4"11"	133
5'	138
5'1"	143
5'2"	147
5'3	152
5'4"	157
5'5"	162
5'6"	167
5'7"	172

Height	Weight
5'8"	177
5'9"	182
5'10"	188
5'11"	193
6'	199
6'1"	204
6'2"	210
6'3"	216
6'4"	221

I weigh **more than** the weight listed for my height.

Yes = 20 No = 0

8 Vegetables

I eat **more than 3 servings** of vegetables a day.

A serving is 1 cup of raw leafy greens or 1/2 cup of other vegetables, cooked or raw.

Yes = 0 No = 25

9 Tomatoes

I eat **5 or more servings** of tomatoes or tomato-based foods like tomato sauce a week.

Yes = 0 No = 15

10 Exercise

I am physically active **less than** 3 hours each week.

Physically active means that you do things like walk, bicycle, run, work around the house, clean or garden. Think about all the time you spend doing these things in a week to get your total hours. Is it less than 3 hours?

Yes = 20 No = 0

Total your points from page 2 here

page 2

Supplemental Fig. 1 Sample page from pen and paper Harvard Cancer Risk Index (1999)

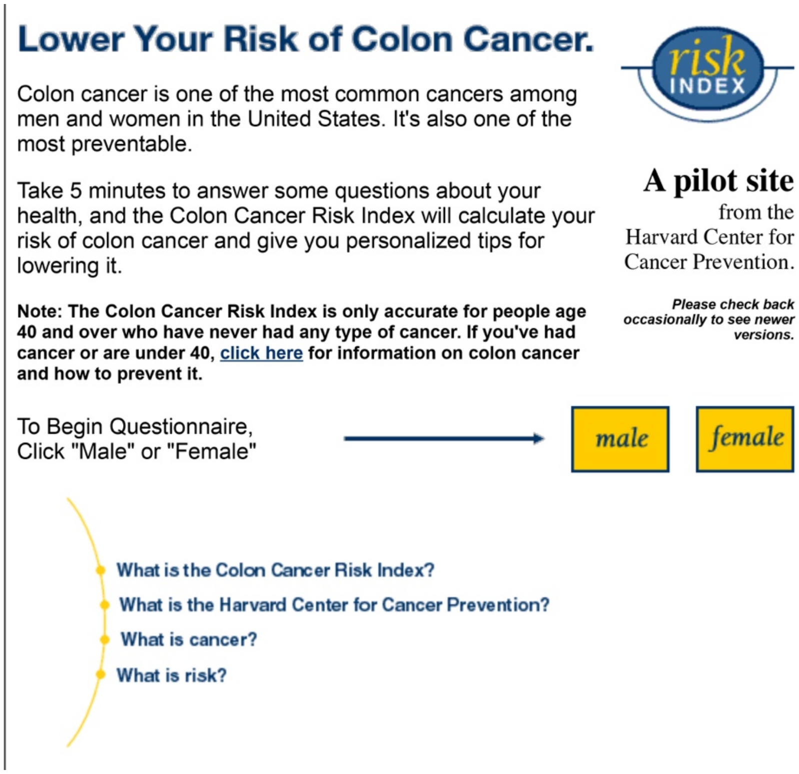

Lower Your Risk of Colon Cancer.

Colon cancer is one of the most common cancers among men and women in the United States. It's also one of the most preventable.

Take 5 minutes to answer some questions about your health, and the Colon Cancer Risk Index will calculate your risk of colon cancer and give you personalized tips for lowering it.

Note: The Colon Cancer Risk Index is only accurate for people age 40 and over who have never had any type of cancer. If you've had cancer or are under 40, click here for information on colon cancer and how to prevent it.

risk INDEX

A pilot site from the Harvard Center for Cancer Prevention.

Please check back occasionally to see newer versions.

To Begin Questionnaire, Click "Male" or "Female"

male

female

What is the Colon Cancer Risk Index?

What is the Harvard Center for Cancer Prevention?

What is cancer?

What is risk?

Supplemental Fig. 2 Home page from Colon Cancer Risk Index pilot (1999)

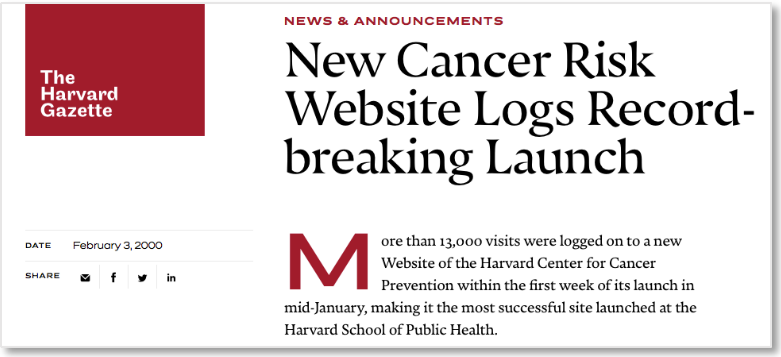

The Harvard Gazette

NEWS & ANNOUNCEMENTS

New Cancer Risk Website Logs Record-breaking Launch

DATE February 3, 2000

SHARE

More than 13,000 visits were logged on to a new Website of the Harvard Center for Cancer Prevention within the first week of its launch in mid-January, making it the most successful site launched at the Harvard School of Public Health.

Supplemental Fig. 3 Harvard Gazette story on initial launch of Your Cancer Risk (2000)

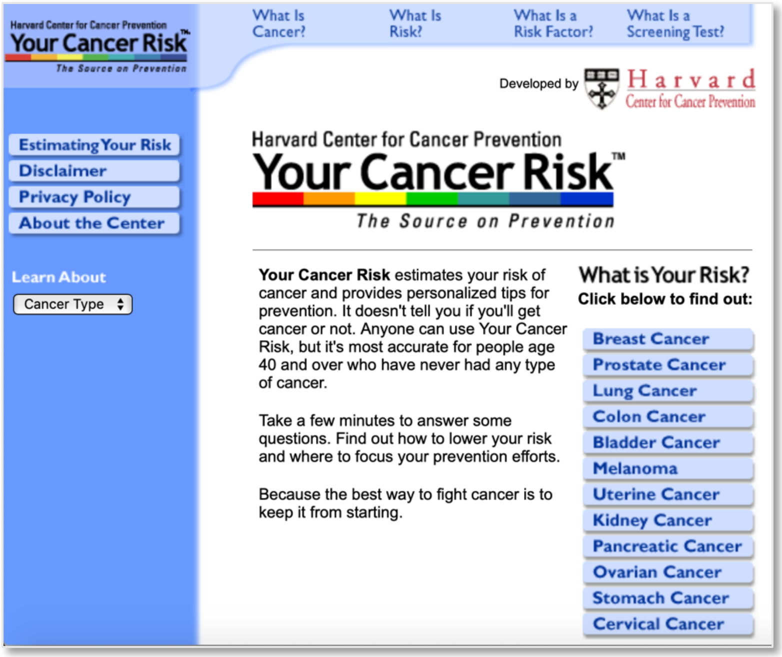

Supplemental Fig. 4 Home page from expanded 12-cancer Your Cancer Risk (2000)

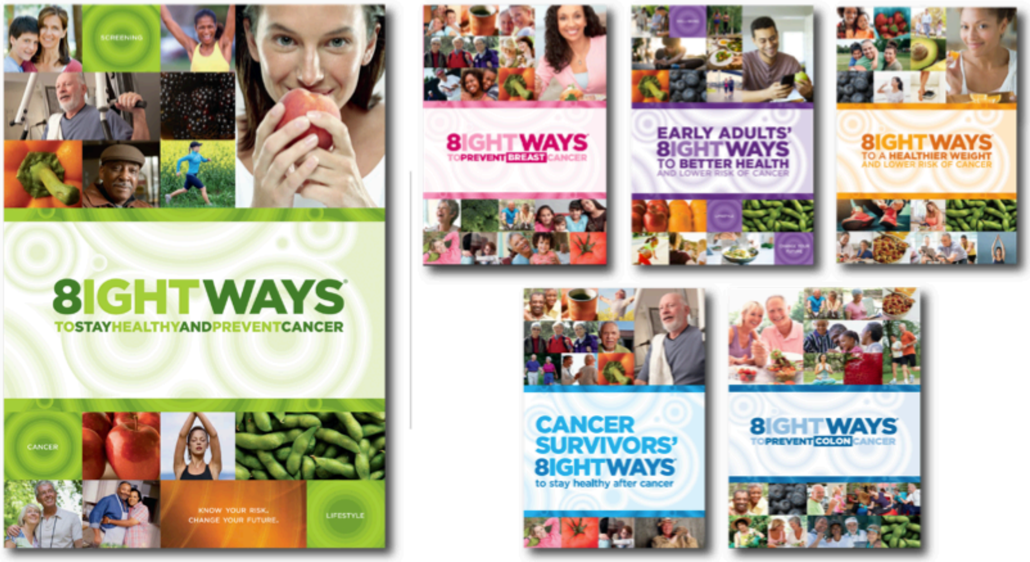

Supplemental Fig. 5 The current 8 Ways health series, which started as part of Your Disease Risk in 2004 (2004 – 2020).

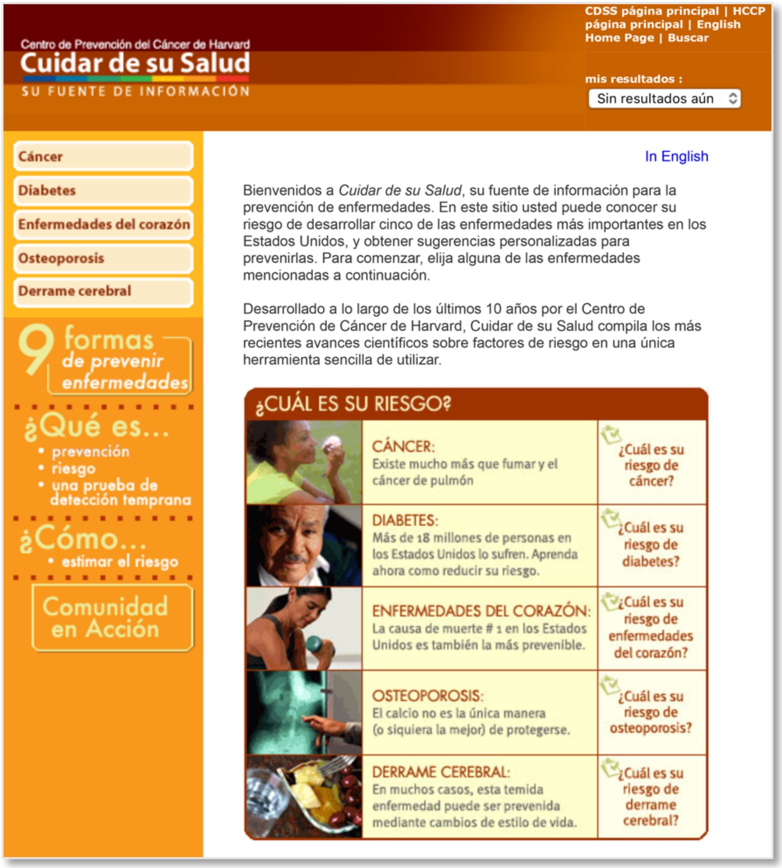

Supplemental Fig. 6 Spanish version of Your Disease Risk: Cuidar de su Salud (2005)

Well

A Better Health Quiz

BY TARA PARKER-POPE MARCH 27, 2009 9:48 AM

More than 27 million people reportedly have taken the RealAge health quiz, which asks questions about lifestyle and family history to determine how young or old your habits make you. But a recent story in The New York Times notes that RealAge often targets its members with health information that may be sponsored by drug marketers.

My problem with the RealAge quiz is its lack of scientific validity. The notion that health behaviors can translate into a meaningful "biological age" is just marketing hype, not real science.

A better health quiz is Your Disease Risk, a useful set of questions to help you determine your risk for diabetes, several cancers, heart disease, osteoporosis and stroke. The quiz can be found on the Web site of the Washington University School of Medicine Siteman Cancer Center. It was created by Dr. Graham Colditz, a noted expert in disease prevention who first developed the quiz with his former colleagues at the Harvard Medical School.

Supplemental Fig. 7 New York Times article, A Better Health Quiz (2009)

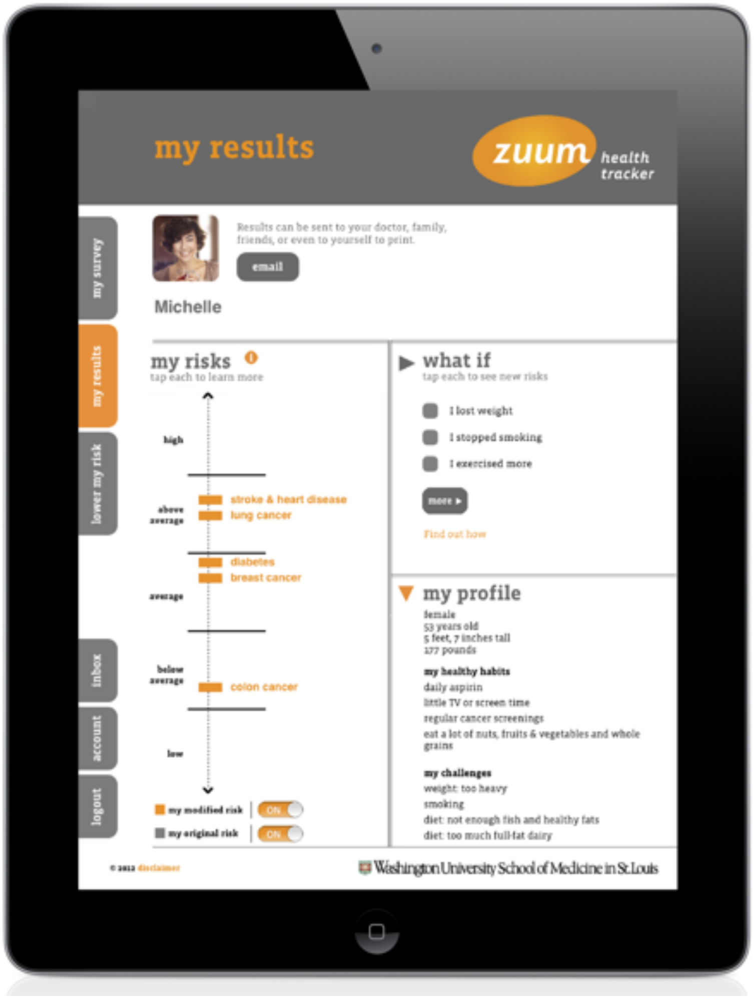

Supplemental Fig. 8 The Zuum iPad app, based on the Your Disease Risk framework (2012)

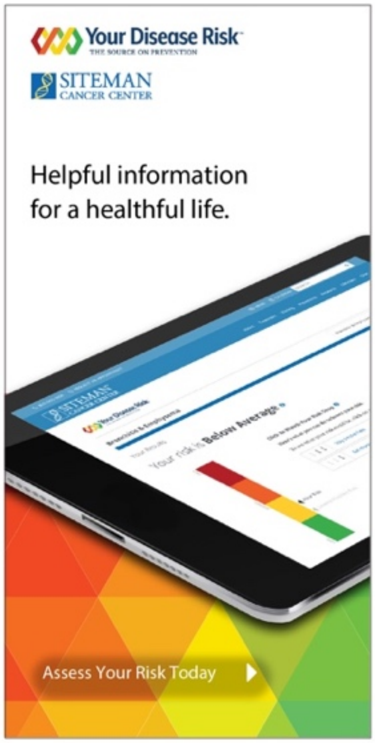

Supplemental Fig. 9 Your Disease Risk ad (2019)
